# Dietary cinnamaldehyde with carvacrol or thymol improves the egg quality and intestinal health independent of gut microbiota in post-peak laying hens

**DOI:** 10.3389/fvets.2022.994089

**Published:** 2022-10-21

**Authors:** Yongshuai Wang, Yilu Wang, Chuanchen Su, Leilei Wang, Xiangyun Lv, Guangya Cui, Longxiang Ji, Yanqun Huang, Huaiyong Zhang, Wen Chen

**Affiliations:** ^1^Key Laboratory of Animal Biochemistry and Nutrition, College of Animal Science and Technology, Ministry of Agriculture, Henan Agricultural University, Zhengzhou, China; ^2^Charoen Pokphand Group Co., Ltd., Zhumadian, China; ^3^Laboratory for Animal Nutrition and Animal Product Quality, Department of Animal Sciences and Aquatic Ecology, Ghent University, Ghent, Belgium

**Keywords:** essential oils, egg quality, intestinal health, microbiota, laying hens

## Abstract

Essential oils have been proven to exert multiple effects on growth performance, production quality, and health status in poultry nutrition, which is dependent on the component and/or dose of essential oils. Diets with the optimal combination of essential oils might be able to improve the performance traits and welfare of laying hens. Therefore, this study was conducted to evaluate the effects of dietary essential oils, which are composed of cinnamaldehyde with carvacrol or thymol, on performance, egg quality, and intestinal health in post-peak laying hens. A total of 384, 50-week-old Hy-line brown laying hens were randomly divided into three groups with 8 replicates of 16 birds each: (1) a basal diet (Ctrl), (2) a basal diet with 100 mg/kg of essential oils consisting of 4.5% cinnamaldehyde with 13.5% carvacrol (CAR+CIN), and (3) a basal diet containing 100 mg/kg of essential oils composed of 4.5% cinnamaldehyde with 13.5% thymol (THY+CIN). The CAR+CIN diet increased the feed consumption from 52 to 55 weeks more than the Ctrl and the THY+CIN diet. Compared with the Ctrl group, the addition of essential oils decreased the dirty egg rate (*P* = 0.07) in the whole trial period. Regarding egg quality, the birds that received the CAR+CIN and THY+CIN diets increased the eggshell strength (*P* = 0.099) or Haugh unit (HU, *p* = 0.03) at 54 weeks, respectively. Supplementation of both CAR+CIN and THY+CIN diets significantly increased the ratio of villus height to crypt depth in the duodenum through increasing villus height and decreasing crypt depth as well as upregulated the mRNA abundances of duodenal *occluding* and *cadherin* (*P* < 0.05). However, the treatment with dietary essential oils did not notably change the proportion of cecal microbiota and bacterial diversity. This study suggested that dietary supplementation of cinnamaldehyde with carvacrol or thymol, the active components of essential oils, could promote egg quality in post-peak laying hens, which might be associated with improved intestinal development and barrier.

## Introduction

The rapid decline in egg production and quality at the end of the laying cycle leads to substantial economic losses (1). Furthermore, the compromised intestinal functions or intestinal flora disturbance due to high-intensity production are also ascribed to the poor egg performance of post-peak laying hens (2). Thus, the strategies to improve the egg production performance, egg quality, and health status during the post-peak period are widely sought out to help extend the laying cycle.

As antibiotics in diets are prohibited globally, aromatic plants and their extracts have been receiving attention due to their positive role in poultry performance and health, in which most of their positive properties mainly contribute to the essential oils and other secondary plant metabolites (3), including cinnamaldehyde, carvacrol, and thymol. Studies on broilers indicated the beneficial roles of 50 mg/kg of cinnamaldehyde in intestinal morphology, microbiota, expressions of nutrient transporters, and apparent ileal digestibility of dry matter and crude protein (4). A linear increase in the ileal digestibility of ash, crude protein, crude fat, calcium, and phosphorus was also observed as dose-dependent supplements of phytogenic feed additive composed of thyme and star anise (0–1,500 mg/kg) in broilers (5). In addition, the diet inclusion of the main active components of essential oils such as carvacrol and cinnamaldehyde can improve growth rate through increased feed intake (FI) due to their food flavor enhancement properties (6). Thymol and carvacrol possess a wide range of biological activities, including antimicrobial activity, modulating immune response, and regulating the gut microbial population (7, 8), and consequently, they are usually used to improve the intestinal development and microbiota composition (e.g., increasing *lactobacilli* and reducing enterococci and *Escherichia coli*) of livestock (9–11). Of note, a synergistic effect between these components might exist. At this point, a combined utilization of thymol and carvacrol was demonstrated to alleviate intestinal inflammation, impair intestinal integrity, and induce barrier dysfunction with *Clostridium perfringens* in broilers (12). The blends of thymol and carvacrol or cinnamaldehyde were also noticed to increase the *Lactobacillus* population in ileal content and modulate the intestinal microbial composition of birds (13, 14). Concerning laying hens, an increasing amount of evidence indicated that diets with 150 mg/kg of essential oils composed of 8% thymol and 4.9% carvacrol improved the percentage of egg production, egg weight, and egg quality of 70-week-old brown laying hens (15). Similarly, the supplementation of 50 mg/kg of essential oils, including 13.5% thymol and 4.5% cinnamaldehyde, in a 53-week-old laying hen diet tended to increase the thickness of the eggshell (16).

Overall, in poultry nutrition, a positive effect of essential oils on growth performance in meat-type poultry has been observed in a limited number of controlled studies and has recently been reviewed by several authors (6, 17). A positive effect of essential oils on performance in laying hens was mostly the result of improved egg quality (15, 18). These roles exerted by essential oils in domestic birds were partly dependent on the component and/or dose of essential oils. In this context, we hypothesized that dietary inclusion of cinnamaldehyde with carvacrol or thymol, the main active components of essential oils, would positively alter the microbial composition and intestinal barrier functions, subsequently conducing the improvements of egg performance and quality in laying hens. Therefore, the objective of this study was to evaluate the effects of dietary cinnamaldehyde with carvacrol or thymol on laying performance, egg quality, microbial community, gut morphology, and relative mRNA expression of tight junction-related genes of laying hens in the late phase of production.

## Materials and methods

### Source of essential oils

Two formulations of essential oils were prepared as powder and provided by Charoen Pokphand Group (Henan, China) with 100 g/ton of feed based on the recommendations of the manufacturer. The carvacrol and cinnamaldehyde essential oils (CAR+CIN) were composed of 13.5% of carvacrol, 4.5% of cinnamaldehyde, 2.0% of holly oil, and 80% of vehicle; the thymus and cinnamaldehyde essential oils (THY+CIN) were composed of 13.5% of thymol, 4.5% of cinnamaldehyde, and 82% of vehicle (Table 1).

**Table 1:** Composition of phytogenic feed additives composed of cinnamaldehyde with thymol or carvacrol essential oils.

**Name**	**Compound**	**Proportion (%)**
CAR+CIN	Carvacrol	13.5
	Cinnamaldehyde	4.5
	Holly oil	2.0
	Vehicle	80.0
THY+CIN	Thymol	13.5
	Cinnamaldehyde	4.5
	Vehicle	82.0

Cinnamaldehyde with carvacrol (CAR+CIN) and cinnamaldehyde with thymol (THY+CIN) represented the basal diet supplemented with 100 mg/kg of cinnamaldehyde with thymol or carvacrol essential oils.

### Experimental design and management

All procedures in the present study were performed in accordance with the Animal Care Committee of Henan Agricultural University. A total of 384 50-week-old Hy-line brown laying hens were randomly assigned to three diet treatments with eight replicates (16 birds each diet) as follows: (1) the basal diet (Ctrl) group, (2) the CAR+CIN diet group (a basal diet with 100 mg/kg of CAR+CIN essential oil), and (3) the THY+CIN diet group (a basal diet with 100 mg/kg of THY+CIN essential oil). After 2 weeks of acclimatization, birds were subjected to the experiment from 52 to 58 weeks of age. Layers were fed two times daily and were given free access to water and the mash experimental diets. The basial diet was designed to satisfy the recommendations of China Agricultural Industry Standards (NY/T 33-2004) for laying hens (19) including 3.6% of calcium and 0.3% of available phosphorus (Table 2). The essential oils were thoroughly mixed with the basal diet. Diets were supplemented in a mashed form to avoid the deactivation of essential oils. Birds were kept in 4-layer vertical cages and housed eight birds per cage. The lighting schedule is 16 h of light and 8 h of darkness, and the average ambient temperature and relative humidity were kept at 23 to 25°C and 30%to 50%, respectively.

**Table 2:** Ingredients and nutrient composition of the basal diet (as-fed).

**Ingredients**	**Proportion (%)**	**Calculated analysis**	**Proportion (%)**
Corn	32.95	AME/(kcal/kg)	2,620
Wheat	30.00	CP	16.30
Soybean meal (46% CP)	12.01	Calcium	3.60
Wheat bran	3.70	Total phosphorus	0.52
Shotcrete corn husk	2.50	Available phosphorus	0.30
Corn gluten powder	1.50	Lysine	0.88
DDGS^a^	3.50	Methionine + cystine	0.67
Sodium chloride	0.24	Threonine	0.61
Limestone	8.60		
CaHPO_4_	0.43		
Chicken bone meal	2.00		
Soybean oil	0.57		
Premix ^b, c^	2.00		
Total	100.0		

^a^DDGS, distillers dried grains with soluble; AME, apparent metabolism energy; CP, crude protein.

^b^Provided per kilogram of diet: Cu (CuSO_4_·5H_2_O), 8 mg; Fe (FeSO_4_·7H_2_O), 80 mg; Zn (ZnSO_4_·7H_2_O), 80 mg; Mn (MnSO_4_·H_2_O), 80 mg; Se (NaSeO_3_), 0.3 mg; I (KI), 0.7 mg; vitamin A, 2,700 IU; vitamin D, 3,400 IU; vitamin E, 10 IU; vitamin K, 0.5 mg; thiamine, 2.0 mg; riboflavin, 5.0 mg; pyridoxine, 3.0 mg; vitamin B_12_, 0.007 mg; calcium pantothenate, 10.0 mg; folate, 0.5 mg; biotin, 0.1 mg; nicotinic acid 30 mg.

^c^The essential oils were supplemented with 0 mg/kg (Ctrl), 100 mg/kg of essential oils composed of 13.5% carvacrol, 4.5% cinnamaldehyde, 2.0% holly oil, and 80% vehicle (CAR+CIN), or 100 mg/kg of essential oils composed of 13.5% thymol, 4.5% cinnamaldehyde, and 82% vehicle (THY+CIN) in basal diets, respectively.

### Egg performance and sampling

Eggs were collected and weighed daily, and the percentage of the egg production was expressed on a hen-day basis during 52 to 55 weeks, 55 to 58 weeks, and overall study intervals. FI was recorded, and the ratio of total FI to total egg weight was calculated for each period. The number of dirty eggs, broken eggs, and unqualified eggs (small eggs, double yolk eggs, soft-shell eggs, and deformed eggs) were counted every day for calculating the percentage of dirty eggs, broken eggs, and misshapen eggs as a percentage of the total number of eggs per week. At the end of the trial, one hen per replicate was randomly selected, weighed, and sampled. The spleen, the pancreas, abdominal fat, and the liver were dissected and weighted, and the relative weight was calculated as follows: fresh weight/final body weight × 100. The numbers of pre-grade yellow follicles (diameter of 6 to 12 mm) and pre-grade white follicles (diameter of 1 to 6 mm) in the ovary were counted. The section of the middle-duodenum was collected and fixed in a 10% neutral buffered formalin solution for the analyses of gut morphology. The mucosa of the duodenum and the cecal content was collected and frozen in liquid nitrogen for intestinal barrier gene expression and microbial resequencing, respectively.

### Egg quality

At 54, 56, and 58 weeks of age, 32 eggs from each treatment were randomly obtained to determine fresh egg quality. After weighing, the width and length of the egg were measured using an electronic digital caliper, and the egg shape index (%) was calculated as (egg width/egg length) × 100. Eggshell strength was determined with an eggshell strength meter (EFG-0503, Robotmation Co., Ltd. Japan). Subsequently, the eggshell was separated, the weight of the eggshell was measured, and relative weight was calculated. The thickness of the eggshell based on the average thickness at the sharp end, the middle part, and the blunt end were determined by the eggshell thickness meter. Haugh unit (HU) and yolk color were measured using an Automatic egg quality tester (EMT-5200, NABEL Co., Ltd. Japan). The height, diameter, and weight of yolk were obtained, and yolk relative weight and yolk index (%) expressed as (yolk height/yolk diameter) × 100 were calculated.

### Crude fat analysis in egg yolks

The crude fat content of each egg was determined based on the standard Association of Official Analytical. Chemists (1995) of method 920.39 (20). Egg yolks were dried for 12 h at 105 ± 3 °C in the oven. Crude fat content was determined using a Soxhlet solvent extraction system. The fat content was calculated from the weight loss after lipid extraction with diethyl ether in the Soxhlet apparatus (Soxtec system HT 1043 extraction unit, Foss Tecator, Sweden). Total extracted fat from egg yolk was expressed as percent fat per 100 g egg yolk.

### Histological analysis of intestine

According to our previous description (21), the formalin-fixed intestinal samples were dehydrated, embedded, sliced into 5-μm transects, and stained with hematoxylin and eosin (H&E), and subsequently, villus height (VH) and crypt depth (CD) of at least 10 well-oriented villi were measured, and the ratio of the villus height to the crypt depth (VH/CD) was calculated. All histomorphometry data acquisition was performed using Image-Pro Plus (IPP 6.0, Cyber Medianetics).

### Gene expression of intestinal barrier

Total RNA was isolated from the mucosa of the duodenum using TRIzol reagent (TransGen Biotech Co. Ltd, Beijing, China). The concentration (ng/μl) and purity (OD 260/280) of the extracted total RNA were determined by Nanodrop 2000 (Thermo Fisher Scientific Inc., USA). After adjusting the concentration of total RNA to 1,000 ng/μl, the RNA was reversely transcribed into cDNA by a commercial kit (Vazyme Biotech Co. Ltd, Nanjing, China). Subsequently, the expression of related genes of the intestinal barrier was determined by using the ABI 7900 fluorescence quantitative PCR system (Applied Biosystems, Warrington, UK) with SYBR qPCR Master Mix (Vazyme Biotech Co. Ltd, Nanjing, China), including zonula occludens-1 (*ZO-1*), mucin-2, *occludin, claudin-1*, and cadherin (*CDH*). The reaction conditions were as follows: 94°C for 5 min, 35 cycles of 94°C for 30 s, 58°C for 30 s, 72°C for 30 s, and a final 10-min extension at 72°C. All the reactions were performed in triplicate. Primers were designed by NCBI online (https://www.ncbi.nlm.nih.gov/) and are shown in Table 3. β-Actin was used as the internal control to normalize the target gene transcript levels.

**Table 3:** The primers for quantitative real-time PCR.

**Gene ID**	**Gene**	**Primer sequences (5' → 3')**	**Product length, bp**
XM_046925214.1	*ZO-1*	F: GAAGAGAGCACAGAACGCAG R: CACTTGTGGCAAGCTGAAGT	123
NM_001013611.2	Claudin-1	F: TCTGGTGTTAACGGGTGTGA R: GTCTTTGGTGGCGTGATCTT	117
NM_205128.1	Occldin	F: CGTTCTTCACCCACTCCTCC R: CCAGAAGACGCGCAGTAAGA	107
NM_001039258.3	*CDH*	F: AGCCAAGGGCCTGGATTATG R: GATAGGGGGCACGAAGACAG	157
NM_001318434.1	Mucin-2	F: AGTGGCCATGGTTTCTTGTC R: TGCCAGCCTTTTTATGCTCT	80
NM_205518.1	β-actin	F: GTCCACCGCAAATGCTTCTAA R: TGCGCATTTATGGGTTTTGTT	78

ZO-1, Zonula occludens-1; CDH, Cadherin.

### Gut microbiome

Total bacterial genomic DNA was extracted from cecal content by the use of the Stool DNA Kits (Qiagen, Hilden, Germany). Then, 16S rRNA genes of distinct regions (16S V4) were amplified using specific primers 515F (GTGCCAGCMGCCGCGGTAA) and 806R (GGACTACHVGGGTWTCTAAT) with unique barcodes. PCR products were mixed in equal density ratios, and the mixed PCR products were purified with Qiagen Gel Extraction Kit (Qiagen, Germany). Sequencing was performed on the Illumina HiSeq platform (Novogene Bioinformatics Technology Co., Ltd, Beijing, China). The obtained sequences were processed for alignment and clustered into operational taxonomic units (OTUs) at 97% similarity using USEARCH (v7.0.1090) in QIIME software. All OTUs were subsequently analyzed for abundance. The alpha diversity was evaluated by calculating the Chao1, Shannon, and Simpson indices. Beta diversity at the genus level was estimated by calculating Bray-Curtis dissimilarity and visualized with principal coordinates analysis (PCoA). Functional inferences were made from the Kyoto Encyclopedia of Gene and Genomes (KEGG) catalog using the Tax4Fun program based on the SILVA database (22).

### Statistical analysis

The data obtained were analyzed by the Kolmogorov-Smirnov and Levene's tests to assess normal distribution and homogeneity of variances, respectively, using SPSS 26 (SPSS Inc., Chicago, IL, USA). A one-way analysis of variance (ANOVA) with Tukey's *post-hoc* test for normal distribution and the Kruskal-Wallis test followed by Dunn's multiple comparisons for non-normal distribution data were performed using the following statistical model:


(1) $$\begin{array}{l} Y_{j}=\mu +D_{j}+\;\varepsilon_{j} \end{array}$$

Where *Y*_*j*_ is the mean value of treatment *j* (Ctrl, carvacrol, or thymol essential oils groups), μ is the overall mean, *Dj* is the fixed effect of treatment *j*, and ε_*j*_ is the error term. The block was further included as a random factor if significant. Significant differences were declared at a *p*-value < 0.05.

## Results

No treatments effects were observed regarding egg weight, FI, and feed conversion ratio (FCR) of laying hens during 52 to 58 weeks (*P* > 0.05), whereas the birds that were fed the CAR+CIN diet exhibited higher FI and the ratio of feed to egg weight during the first 1 to 3 weeks as compared to those that received the THY+CIN diet (*P* < 0.01; Table 4). There were also no obvious effects (*P* > 0.05) of dietary treatments on the body weight of the bird and the relative weight of organs of post-peak laying hens (Table 5).

**Table 4:** Effects of dietary treatments on egg production of the post-peak laying hens.

**Items**	**Ctrl**	**CAR+CIN**	**THY+CIN**	***P-*value**
**Egg mass, g/bird/day**
52–55 weeks	59.49 ± 0.45	59.75 ± 0.25	59.42 ± 0.22	0.742
55–58 weeks	60.06 ± 0.41	60.38 ± 0.38	59.93 ± 0.23	0.649
52–58 weeks	59.77 ± 0.42	60.07 ± 0.29	59.68 ± 0.22	0.673
**Feed intake, g/bird/day**
52–55 weeks	113.41 ± 0.96 ^ab^	116.34 ± 0.86^a^	111.07 ± 1.11^b^	0.004
55–58 weeks	117.46 ± 0.85	117.76 ± 0.86	117.19 ± 1.89	0.953
52–58 weeks	115.43 ± 0.88	117.05 ± 0.84	114.13 ± 1.34	0.167
**Feed to egg mass ratio, g feed/g egg**
52–55 weeks	2.09 ± 0.02^ab^	2.15 ± 0.03^a^	2.02 ± 0.02^b^	0.005
55–58 weeks	2.13 ± 0.03	2.11 ± 0.03	2.10 ± 0.03	0.707
52–58 weeks	2.11 ± 0.02	2.12 ± 0.01	2.10 ± 0.02	0.743

In this study, a total of 384 50-week-old Hy-line brown laying hens were randomly divided into a basal diet (Ctrl), a basal diet with 100 mg/kg of essential oils composed of 4.5% of cinnamaldehyde with 13.5% of carvacrol (CAR+CIN), and a basal diet consisting 100 mg/kg of essential oils composed of 4.5% cinnamaldehyde with 13.5% thymol (THY+CIN) for 6 weeks.

Values are mean ± standard error (n = 8). Different letters in the same column represent the significant difference (P < 0.05).

**Table 5:** Effects of dietary treatments on body weight and organ size of post-peak laying hens.

**Items**	**Ctrl**	**CAR+CIN**	**THY+CIN**	***P-*value**
Body weight, kg	1.87 ± 0.14	1.93 ± 0.12	1.96 ± 0.18	0.445
Relative spleen weight, %	0.12 ± 0.03	0.13 ± 0.03	0.12 ± 0.03	0.686
Relative pancreases weight, %	0.17 ± 0.02	0.17 ± 0.03	0.17 ± 0.01	0.919
Relative abdominal fat weight, %	2.72 ± 1.55	2.75 ± 0.86	2.80 ± 1.14	0.992
Relative liver weight, %	1.77 ± 0.07	1.63 ± 0.26	1.71 ± 0.20	0.355

In this study, a total of 384 50-week-old Hy-line brown laying hens were randomly divided into a basal diet (Ctrl), a basal diet with 100 mg/kg of essential oils consisting of 4.5% cinnamaldehyde with 13.5% carvacrol (CAR+CIN), and a basal diet containing 100 mg/kg of essential oils composed of 4.5% cinnamaldehyde with 13.5% thymol (THY+CIN) for 6 weeks.

Values are mean ± standard error (n = 8). A P-value < 0.05 is regarded as a difference significantly.

**Figure 1:**
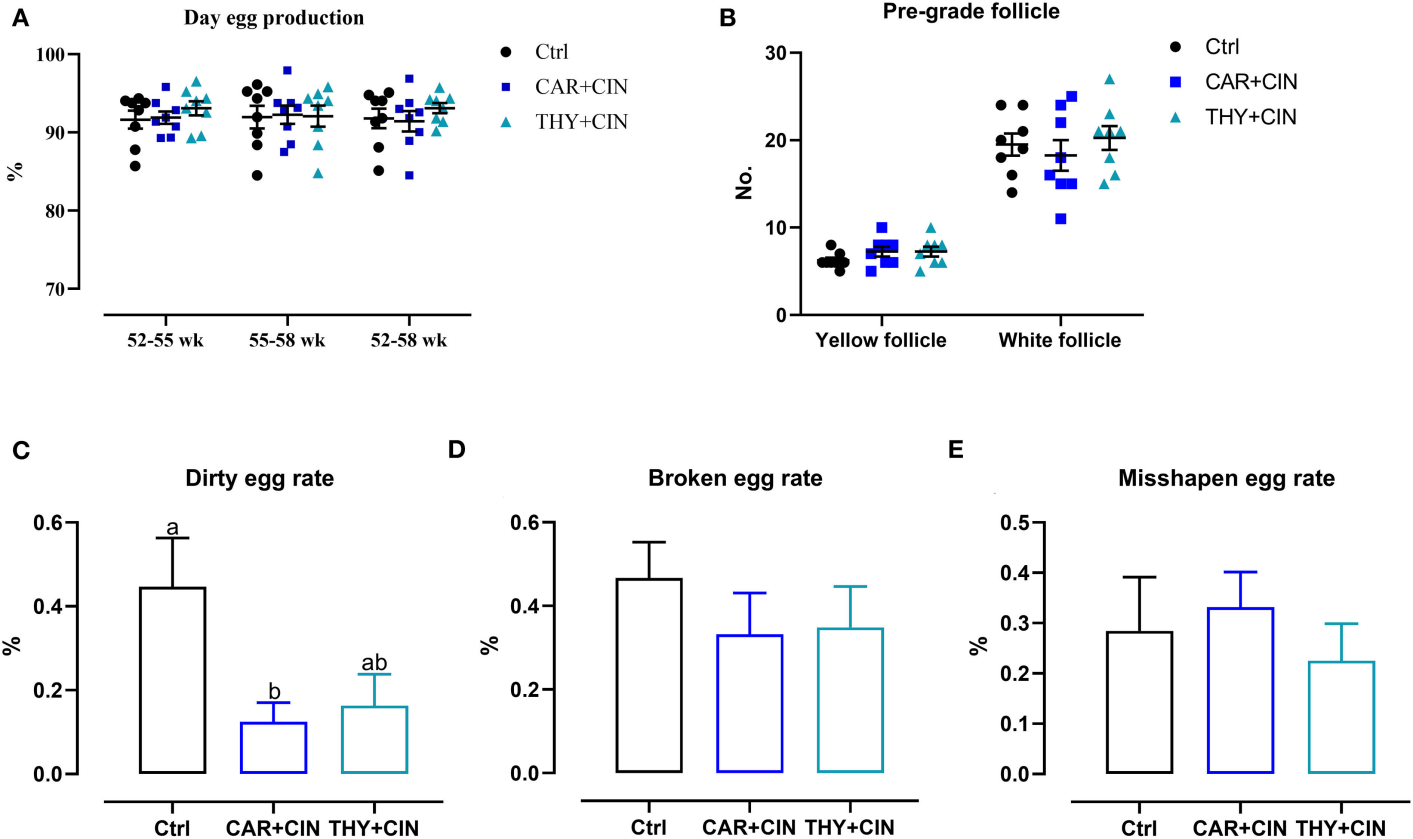
The effects of dietary cinnamaldehyde with carvacrol (CAR+CIN) or thymol (THY+CIN) essential oils on **(A)** day egg production. **(B)** Number of the pre-grade follicle. **(C)** Dirty egg rate. **(D)** Broken egg rate. **(E)** Misshapen egg rate of post-peak laying hens. Means without common letter are significantly different (*P* < 0.05, *n* = 8). Yellow follicles were pre-grade yellow follicles in the range of 6 - 12 mm in size in the ovary; white follicles were pre-grade white follicles in the range of 1–6 mm in size in the ovary. Dirty eggs, broken eggs, and misshapen eggs percentage were calculated as a percentage of the total number of eggs per week. In this study, a total of 384, 50-week-old Hy-line brown laying hens were randomly divided into a basal diet (Ctrl), a basal diet with 100 mg/kg of essential oils consisting of 4.5% cinnamaldehyde with 13.5% of carvacrol (CAR+CIN), and a basal diet containing 100 mg/kg of essential oils composed of 4.5% of cinnamaldehyde with 13.5% of thymol (THY+CIN) for 6 weeks.

Dietary treatment did not affect the laying rate and the number of the pre-grade yellow follicle during the whole experiment period (*P* > 0.05; Figures 1A,B). When compared with the Ctrl group, the dirty egg rate was decreased by 0.32% and 0.29% in the CAR+CIN and THY+CIN diets, respectively (Figure 1C). However, the broken egg rate and the misshapen egg rate were comparable among experimental groups (*p* > 0.05, Figures 1D,E).

### Egg quality response to essential oil supplementation

The results of egg quality are presented in Table 6. No significant alterations (*P* > 0.05) were observed in terms of egg weight, egg shape index, eggshell properties, yolk color, and the relative weight of yolk, and the crude fat content in egg yolk responded to the supplementation of dietary essential oils (Table 6). Compared with the Ctrl group, the eggshell strength of layers that were fed the CAR+CIN diet showed an increasing trend at 54 weeks (*P* = 0.099). In addition, the HU was notably higher in the THY+CIN diet than.

**Table 6:** Effects of dietary treatments on egg quality of post-peak laying hens.

**Items**	**Ctrl**	**CAR+CIN**	**THY+CIN**	***P-*value**
Egg mass, g
54 weeks	60.42 ± 0.57	60.66 ± 0.70	60.40 ± 0.57	0.968
56 weeks	59.67 ± 0.34	61.53 ± 0.78	60.10 ± 0.63	0.104
58 weeks	60.53 ± 0.43	59.27 ± 0.55	59.08 ± 0.62	0.147
Egg shape index
54 weeks	1.30 ± 0.01	1.30 ± 0.01	1.29 ± 0.01	0.465
56 weeks	1.30 ± 0.01	1.29 ± 0.01	1.30 ± 0.01	0.214
58 weeks	1.31 ± 0.01	1.29 ± 0.01	1.31 ± 0.01	0.160
Eggshell properties
Strength, N
54 weeks	44.28 ± 1.08	47.78 ± 1.46	45.40 ± 0.62	0.099
56 weeks	43.91 ± 0.97	46.08 ± 1.31	44.30 ± 0.94	0.184
58 weeks	43.56 ± 0.58	44.39 ± 1.36	43.27 ± 1.22	0.824
Thickness, mm
54 weeks	0.34 ± 0.005	0.34 ± 0.004	0.34 ± 0.004	0.999
56 weeks	0.32 ± 0.003	0.33 ± 0.006	0.33 ± 0.005	0.327
58 weeks	0.32 ± 0.005	0.32 ± 0.005	0.33 ± 0.006	0.539
Relative weight, %
54 weeks	13.41 ± 0.44	13.60 ± 0.19	13.65 ± 0.14	0.553
56 weeks	14.05 ± 0.13	14.05 ± 0.26	13.82 ± 0.17	0.637
58 weeks	13.61 ± 0.38	13.16 ± 0.61	13.33 ± 0.24	0.306
Yolk properties
Relative weight, %
54 weeks	27.64 ± 0.32	27.56 ± 0.30	27.90 ± 0.43	0.784
56 weeks	27.13 ± 0.28	26.45 ± 0.38	27.12 ± 0.32	0.271
58 weeks	26.32 ± 0.29	27.28 ± 0.38	27.47 ± 0.43	0.086
Color
54 weeks	8.17 ± 0.10	8.22 ± 0.05	8.12 ± 0.06	0.611
56 weeks	8.38 ± 0.07	8.23 ± 0.77	8.47 ± 0.10	0.141
58 weeks	8.35 ± 0.08	8.30 ± 0.06	8.40 ± 0.04	0.529
Crude fat Content in egg yolk, %
58 weeks	60.73 ± 1.62	66.04 ± 0.83	65.23 ± 2.75	0.100
Haugh unit
54 weeks	78.70 ± 2.48^b^	74.07 ± 1.65^b^	82.21 ± 1.77^a^	0.030
56 weeks	85.90 ± 0.83	85.38 ± 0.93	86.20 ± 0.63	0.768
58 weeks	84.59 ± 0.61	84.56 ± 1.04	83.58 ± 0.65	0.598

In this study, a total of 384 50-week-old Hy-line brown laying hens were randomly divided into a basal diet (Ctrl), a basal diet with 100 mg/kg of essential oils consisting of 4.5% of cinnamaldehyde with 13.5% of carvacrol (CAR+CIN), and a basal diet containing 100 mg/kg of essential oils composed of 4.5% cinnamaldehyde with 13.5% thymol (THY+CIN) for 6 weeks.

Values are mean ± standard error (n = 8). Different letters in the same column represent the significant difference (P < 0.05).

(*P* = 0.030) that of the Ctrl and the CAR+CIN diet at 54 weeks (Table 6).

### Dietary essential oils improved intestine development and barrier

**Figure 2:**
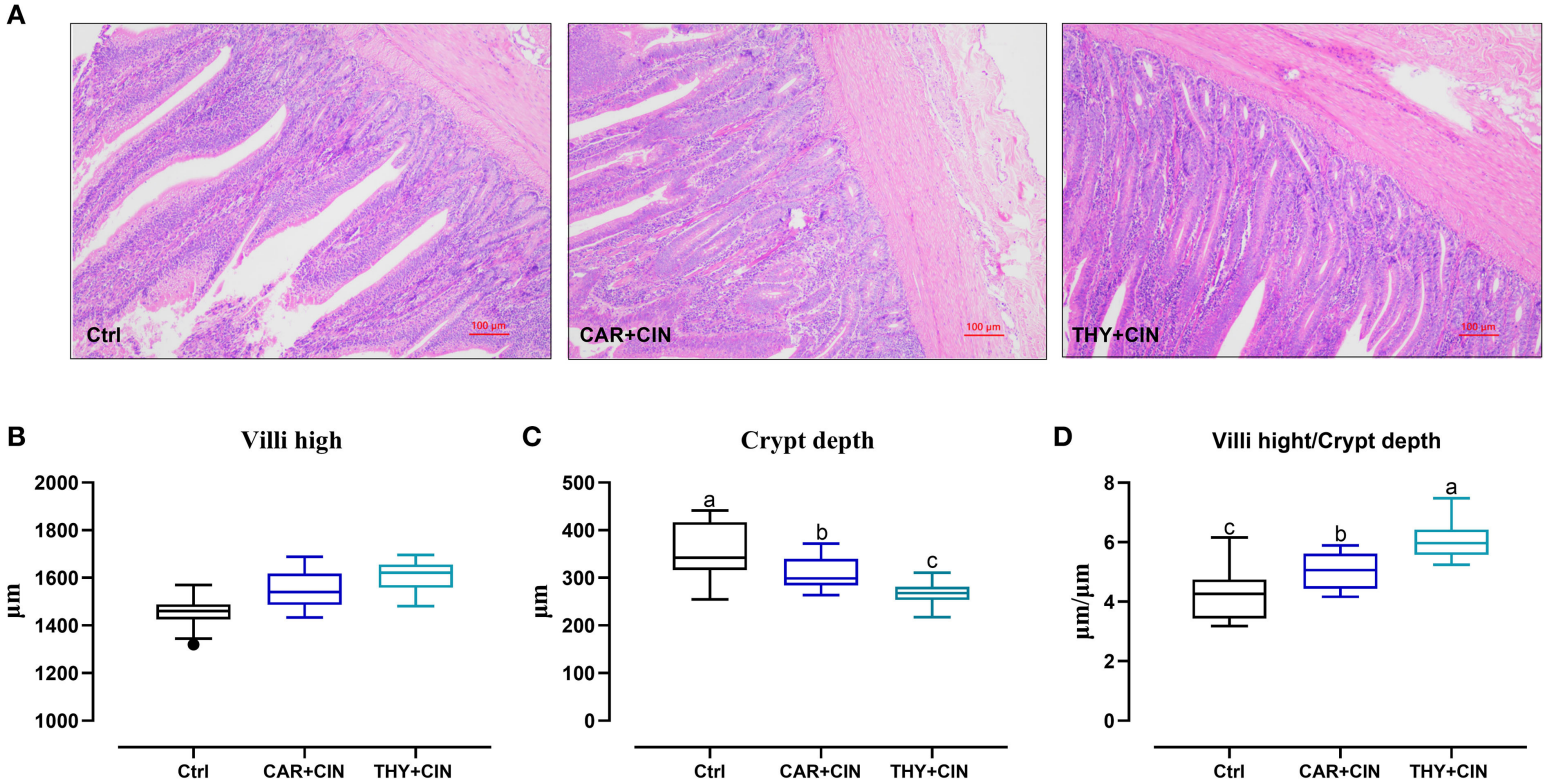
The effects of dietary cinnamaldehyde with carvacrol (CAR+CIN) or thymol (THY+CIN) essential oils on the duodenal structure of post-peak laying hens. **(A)** Representative hematoxylin/eosin staining of the duodenum and **(B)** villus height. **(C)** Crypt depth. **(D)** The ratio of villus high to crypt depth were measured in the duodenum. Scale bar = 100 μm. Boxes are bounded by the 25th and 75th percentiles with the median shown by the line bisecting the box in the box-whiskers plot. Whiskers extend to the full range of the data. Outliers are represented by dots. Means without a common letter are significantly different (*P* < 0.05). In this study, a total of 384 50-week-old Hy-line brown laying hens were randomly divided into a basal diet (Ctrl), a basal diet with 100 mg/kg of essential oils consisting of 4.5% cinnamaldehyde with 13.5% of carvacrol (CAR+CIN), and a basal diet containing 100 mg/kg of essential oils composed of 4.5% of cinnamaldehyde with 13.5% of thymol (THY+CIN) for 6 weeks.

**Figure 3:**
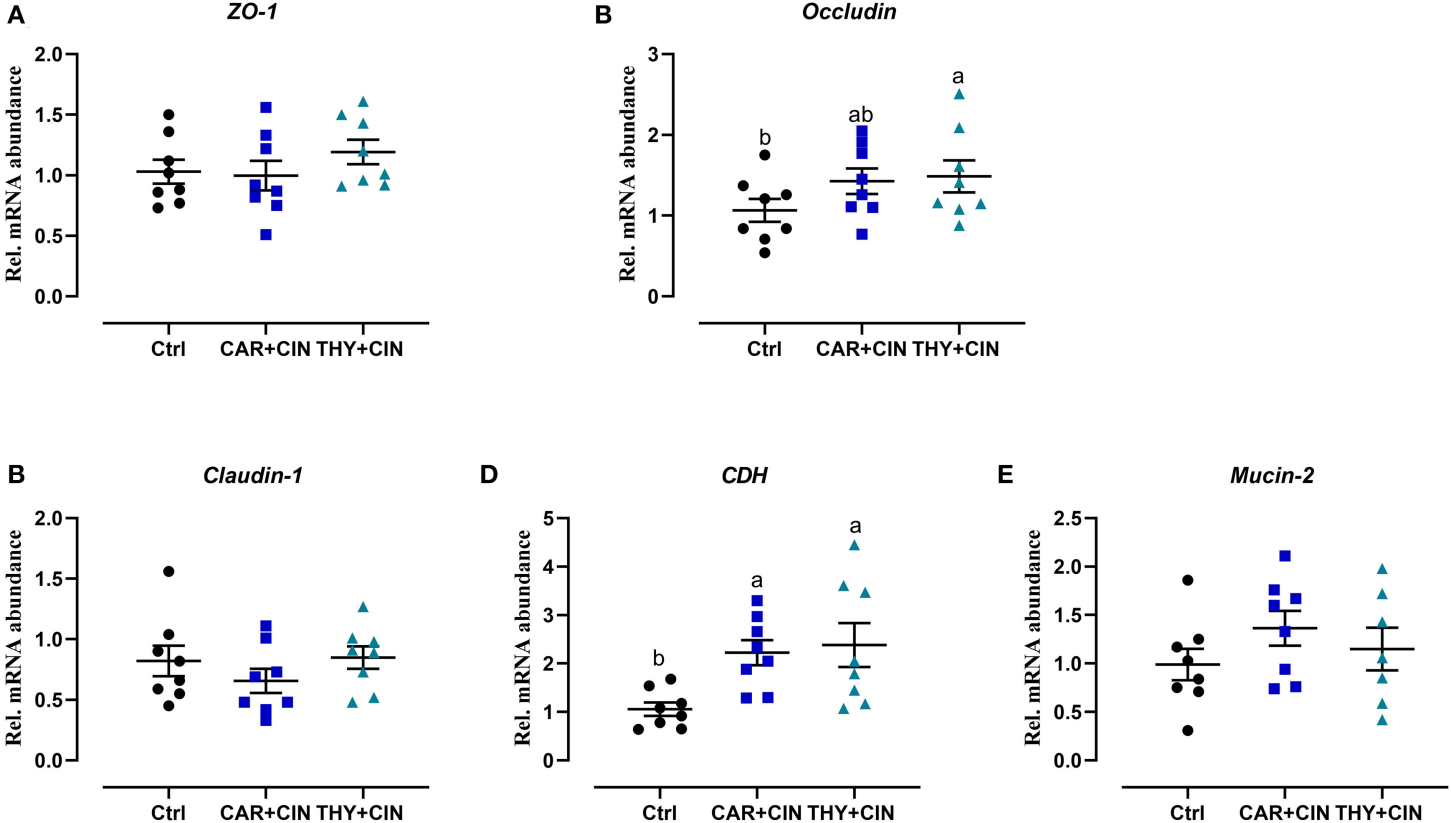
The effect of dietary cinnamaldehyde with carvacrol (CAR+CIN) or thymol (THY+CIN) essential oils on mRNA levels related to the intestinal barrier in mid-duodenum of post-peak laying hens. Tight junction protein including **(A)** Zonula occludens-1 *(ZO-1)*. **(B)** *Occludin*. **(C)** *Claudin-1*. **(D)** Cadherin (*CDH*). **(E)** *Mucin-2* mRNA abundance. Values are mean ± standard error in scatter plot (*n* = 8). Means without a common letter are significantly different (*P* < 0.05). In this study, a total of 384, 50-week-old Hy-line brown laying hens were randomly divided into a basal diet (Ctrl), a basal diet with 100 mg/kg of essential oils consisting of 4.5% of cinnamaldehyde with 13.5% of carvacrol (CAR+CIN), and a basal diet containing 100 mg/kg of essential oils composed of 4.5% of cinnamaldehyde with 13.5% of thymol (THY+CIN) for 6 weeks.

Intuitively, the treatment of dietary essential oils improved intestine integrity and resulted in a well-oriented structure (Figure 2A). Diets with essential oils had a significant effect (*P* < 0.05) on intestinal development of the duodenum, evidenced by decreased CD and increased VH/CD values (Figures 2B–D). As far as the duodenal barrier was concerned, the dietary treatments did not significantly change (*P* > 0.05) the mRNA level of *ZO-1, claudin-1*, and *mucin-2* in the duodenum (Figure 3). However, when compared to the Ctrl group, the THY+CIN diet significantly increased the expression of duodenal *occludin* (*P* < 0.05; Figure 3B), and the supplementation of both CAR+CIN and THY+CIN diets notably upregulated the transcription of *CDH* in the duodenum (*p* < 0.05; Figure 3D).

### Dietary essential oils failed to alter cecal microbiota

**Figure 4:**
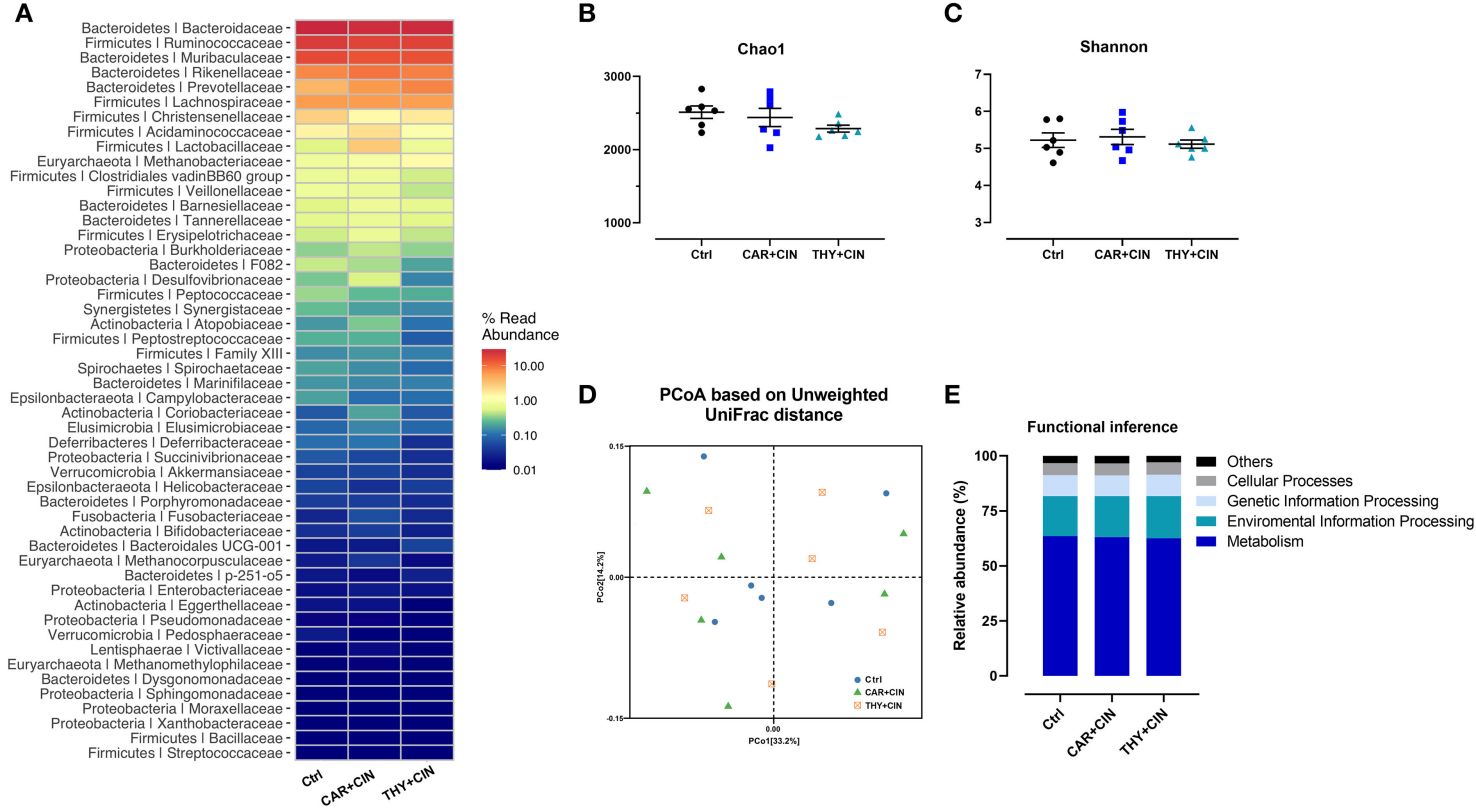
The effect of dietary cinnamaldehyde with carvacrol (CAR+CIN) or thymol (THY+CIN) essential oils on cecal microbiota. **(A)** The relative abundances of bacterial communities at the family level; **(B,C)** Chao1, and Shannon indicesexes were used to assess diversity and evenness at the familyiarlevel, **(D)** principal coordinate analysis plot (PCoA) of caecum microbiome diversity based on Unweighted UniFrac distance. **(E)** Functional predictions for the cecal microbiome based on Kyoto Encyclopedia of Genes and Genomes (KEGG) analysis. Significant differences were declared at a *P*-value < 0.05. In this study, a total of 384 50-week-old Hy-line brown laying hens were randomly divided into a basal diet (Ctrl), a basal diet with 100 mg/kg of essential oils consisting of 4.5% of cinnamaldehyde with 13.5% of carvacrol (CAR+CIN), and a basal diet containing 100 mg/kg of essential oils composed of 4.5% of cinnamaldehyde with 13.5% of thymol (THY+CIN) for 6 weeks.

No significant differences (*P* > 0.05) in the relative abundance of species and the richness were observed in cecal microbiota at the family level, which are evidenced by similar Chao1 and Shannon indices (Figures 4A–C). The PCoA results also showed no discernible separation of cecal microbial communities between Ctrl and essential oils-supplemented groups (*P* > 0.05, Figure 4D). The functional inference analysis was conducted, and it indicated that the main metabolic pathways of the functional genes in the microbial community were not influenced by the dietary supplementation of essential oils, including metabolism, environmental information processing, genetic information processing, and cellular processes (Figure 4E).

## Discussion

During the late phase of the laying cycle, several problems including decreased performance traits, inferior egg quality, and compromised intestinal barrier are paramount and increasing due to the growing concern over the security of human food and animal welfare. Some active components of essential oils, such as cinnamaldehyde, carvacrol, and thymol, have been recognized as important nutrients for improving the performance, intestinal integrity, and microbiota of broilers (5, 12) and laying hens (15, 23). In the present study, the dietary supplementation of cinnamaldehyde with carvacrol or thymol contributed to the improvement of egg quality and intestinal barrier of post-peak laying hens, which provided evidence of using the blend of cinnamaldehyde and carvacrol or thymol as a potential feed additive for laying hens.

Recent studies showed that essential oils composed of cinnamaldehyde, carvacrol, and thymol are suitable to be used as growth promoters in broilers, which increased the weight gain and final body weight and decreased the ratio of feed to gain (24–26). The outcomes from laying hens found that dietary supplementation with a mixture of 13.5% of thymol and 4.5% of cinnamaldehyde could increase egg production and FCR (16). Nevertheless, there was abundant evidence indicating that the diet with carvacrol or thymol does not significantly promote the egg mass, FI, and FCR of laying hens (27, 28), which was further supported by our current study. In the present study, dietary cinnamaldehyde with carvacrol or thymol supplementation did not significantly influence the egg mass, egg production, FI, and FCR of laying hens between 55 to 58 weeks and 52 to 58 weeks. The FI and the ratio of feed to egg weight during 52 to 55 weeks were notably increased by the administration of the CAR+CIN diet. The increase in feed consumption in the early phase might be due to the food flavor enhancement properties of essential oils (6). It was reported that the essential oils extracted from herbs could improve the palatability of feed and feed consumption of swine and poultry due to their aromatic characteristics (29). For the laying rate, the comparable egg production could be explained by the similar number of yellow and white follicles in this study. In addition, the frequency of egg defects (such as dirty eggs, broken eggs, and malformed eggs) in commercial layer chickens has substantially threatened the egg quality and food security for humans. From this perspective, the reduced rate of dirty eggs in the current study suggested the positive role of dietary cinnamaldehyde with carvacrol or thymol on performance traits of post-peak laying hens.

Egg quality defines those characteristics of an egg that affect consumer acceptability and preference. Findings from previous experiments indicated that dietary supplementation of 150 mg/kg of essential oils composed of 8% of thymol and 4.9% of carvacrol improved the external and internal quality of the egg of 70-week-old brown laying hens (15). On the other hand, the dietary supplementation with oregano essential oil at levels of 50 and 100 mg/kg showed no significant differences in egg weight and shape of the egg, the size and color of the yolk, HU, and shell thickness (30). Dietary microencapsulated oregano supplementation also failed to change the relative weight of eggshell, shape index, albumen height, HU, and yolk color at the end of weeks 4, 8, and 12 in laying hens (2). In this study, supplementation of essential oils did not significantly increase the egg weight, egg shape index, eggshell properties, yolk color, the relative weight of yolk during the whole trial period and the crude fat content in egg yolk at 58 weeks. This variability in results might be related to the dose and components of essential oils, as well as their metabolism and utilization in domestic birds. Moreover, we noticed that the increase in eggshell strength of 53-week-old layers received the CAR+CIN diets, which was consistent with the observations from the previous study, showing that the addition of essential oil mixtures increased shell breaking strength (28) and that dietary supplementation with a blend of 13.5% of thymol and 4.5% of cinnamaldehyde enhanced the eggshell strength (16). This is partly attributed to the fact that essential oil could positively influence the mineral absorption rate, especially that of calcium and magnesium ions (31, 32). The enhancement of eggshell strength might be a critical factor to reduce the broken egg rate in this study. Cabuk et al. (2006) reported that dietary supplementation of essential oil mixture including cinnamaldehyde, carvacrol, and thymol decreased the cracked-broken egg rate when compared with the control diet (33). Moreover, it is well-known that the HU is a measure of egg protein quality based on the height of its egg white (albumen). In this study, the supplementation of dietary THY+CIN significantly increased the HU of the egg from post-peak laying hens. Taken together, the supplementation of the CAR+CIN and THY+CIN diets in laying hens shows a positive effect on egg quality by enhancing the strength of eggshells and increasing HU, respectively.

Disruption of tight junctions and microbiota dysbiosis due to long-term egg production may be partially responsible for the lower nutrient absorption and the compromised laying performance of laying hens in the late production period (34). The intestinal barrier formed by epithelial cell tight junctions and the mucus and the gut microbiota that covers its surface play vital roles in permitting the absorption of nutrients, electrolytes, and water, which acts as a selectively permeable barrier, while maintaining an effective defense layer against intraluminal toxins, antigens, and enteric flora (35), indicating that the enhancement of intestinal barrier function may occur during the improvement of egg performance and egg quality in the present study. Carvacrol and thymol are commonly used to improve the villus structures and microbiota composition, due to their antimicrobial and antioxidative characteristics, to strengthen the gut health of livestock (9–11). However, an optimal combination of carvacrol, thymol, and cinnamaldehyde might promote the intestinal health of laying hens. The results of this study showed that the addition of cinnamaldehyde with carvacrol or thymol notably upregulated the expression of *occludin* and *CDH* in the duodenum, both of which were key tight junction proteins to maintaining intestinal integrity. Combined utilization of thymol and carvacrol was homoplastically demonstrated to alleviate the impaired intestinal integrity and barrier dysfunction induced by *C. perfringens* challenge in broilers (12). Furthermore, we also confirmed that dietary supplementation of 100 mg/kg of cinnamaldehyde with carvacrol or thymol enhanced duodenal VH/CD by increasing VH and declining CD. This result was consistent with the results of He et al. (23), who reported that a diet supplemented with 100 mg/kg of oregano essential oils including carvacrol and thymol increased duodenum VH in 30-week-old Hy-Line Layers (23). The length of the intestinal villi determines the surface area of intestinal nutrient absorption, and the intestinal crypt is correlated with tissue turnover. A shortening of the villus and a large crypt could lead to poor nutrient absorption, increased secretion in the gastrointestinal tract, diarrhea, reduced disease resistance, and lower overall performance (36, 37). Therefore, any improvement in the intestinal structure and barrier contributed by the dietary supplementation of cinnamaldehyde with carvacrol or thymol might facilitate the absorption of nutrients and consequently improve the egg quality in this study. To support this, the apparent ileal digestibility of crude ash, crude protein, crude fat, calcium, and phosphorus showed a linear increase with the supplementation of essential oils (containing thymol and anethole as lead active components) in broilers (5). The addition of 100 or 200 mg/kg of essential oils (including carvacrol and thymol as lead active components) was also found to increase the dry matter, organic matter, and crude protein digestion when compared to the control group (38).

To better understand the mechanisms linking dietary essential oils and performance traits of laying hens, further analysis was conducted on gut microbiota, whose interactions with the gut play a crucial role in the prevention of pathogen colonization, maintenance of immune homeostasis, and metabolism of nutrients. Given the fact that cinnamaldehyde, carvacrol, and thymol in feed promoted the growth of beneficial bacteria, such as *Lactobacillus*, and inhibited the growth of potentially harmful intestinal bacteria, including *Escherichia coli* and *C. perfringens* (13, 14, 39), the alterations in the composition of gut microbiota due to the administration of dietary essential oils are expected in the present study. The outcomes from the cecal microbiota composition showed that treatment with essential oils did not notably change the proportion of cecal microbiota, evidenced by alpha and beta diversities. The functional inference analysis also revealed that the main metabolic pathways of the functional genes in the microbial community were not influenced by the supplementation of dietary essential oils in our study. Indeed, one unique feature of essential oils is their hydrophobicity, rendering the ability to react with lipids on the bacterial cell membrane, which increases the membrane permeability, disturbs the original cell structures, and breaks homeostasis (40). Family analysis in the late-phase laying hens indicated that the abundance of *Bifidobacteriaceae* tended to increase with essential oil addition, and the proportion of *Aeriscardovia* and *Aquabacterium* increased at the genus level (2). Similarly, the number of *Campylobacter* was reduced with a 1% carvacrol addition in the feed of broilers (41). Collectively, further research is needed to evaluate the possibility of essential oil, inducing the alteration in the cecal microbiota of post-peak laying hens.

## Conclusion

In conclusion, this study demonstrated that dietary supplementation of cinnamaldehyde with carvacrol or thymol, the active components of essential oils, could promote egg quality by enhancing the strength of eggshell or HU in post-peak laying hens, which could be in part responsible for improved intestinal development and barrier. These findings may provide insights into the underlying rationalization of essential oils utilized in laying hens.

## Data availability statement

The datasets presented in this study can be found in online repositories. The names of the repository/repositories and accession number(s) can be found in the article/supplementary material.

## Ethics statement

The animal study was reviewed and approved by Institutional Animal Care and Use Committee at the Henan Agricultural University.

## Author contributions

YoW, CS, and LW: data curation and writing—original draft preparation. YH: investigation and formal analysis. YiW: methodology. HZ, XL, and WC: review and correction. WC: supervision and writing—review and editing. All authors contributed to the article and approved the submitted version.

## Funding

This work was supported by the fund from the National Natural Science Foundation of China (No. 32072748) and the Innovative Leading Talents Project of Zhengzhou (201845).

## Conflict of interest

Authors XL, GC, and LJ were employed by the company Charoen Pokphand Group Co., Ltd. The remaining authors declare that the research was conducted in the absence of any commercial or financial relationships that could be construed as a potential conflict of interest.

## Publisher's note

All claims expressed in this article are solely those of the authors and do not necessarily represent those of their affiliated organizations, or those of the publisher, the editors and the reviewers. Any product that may be evaluated in this article, or claim that may be made by its manufacturer, is not guaranteed or endorsed by the publisher.
